# Study on the sedative effect and safety of oral midazolam combined with dexmedetomidine nasal drops in children during magnetic resonance imaging examination

**DOI:** 10.3389/fped.2024.1500277

**Published:** 2025-01-06

**Authors:** Yuancui Li, Rongzhu Lei

**Affiliations:** Department of Anesthesiology, Shanxi Children’s Hospital, Taiyuan, China

**Keywords:** midazolam, dexmedetomidine, children, MRI, sedative effectiveness, safety

## Abstract

**Background:**

Magnetic resonance imaging (MRI) is a crucial non-invasive diagnostic tool for pediatric diseases, requiring patients to remain still, often with the use of sedatives. Midazolam and dexmedetomidine are commonly used for sedation in children, but their combined effect needs further study. This study aims to evaluate the safety and effectiveness of combining intranasal dexmedetomidine (ID) with oral midazolam (OM) in children undergoing MRI, and assess its clinical feasibility.

**Methods:**

A prospective, randomized controlled trial was conducted with 196 pediatric patients undergoing MRI from January 2022 to December 2023. Patients were randomly assigned to a control group (OM alone) or an observation group (OM + ID), with 98 cases each. Total sedation time, wake-up time, onset time, and adverse reactions were evaluated. Sedation effectiveness was assessed using the Ramsay Sedation Score.

**Results:**

The observation group had significantly longer total sedation time (*P* = 0.039) and higher one-time sedation success rate (*P* = 0.038) compared to the control group. The Ramsay score indicated better sedation effects in the observation group (*P* < 0.05). Adverse events were similar between groups and resolved with rest.

**Conclusion:**

Combining ID with OM provides effective sedation for pediatric MRI, with an acceptable safety profile, supporting its use in clinical practice.

## 1 Introduction

Accurate diagnosis and successful treatment of pediatric disorders need the use of high-quality medical imaging equipment. Magnetic resonance imaging (MRI) has become a key technology in this regard due to its high resolution and sensitivity to various tissue types (1, 2). However, pediatric MRI requires children to remain still for extended periods, which can be challenging due to their age and emotional state. Therefore, effective sedation strategies are essential for ensuring optimal image quality and minimizing the need for repeat examinations (3). Given the high cost of general anesthesia, sedation-assisted MRI has become a preferred approach in clinical practice. Consequently, the development and refinement of safe and efficient sedation protocols have emerged as a significant area of research (4, 5).

Chloral hydrate and propofol are commonly used sedatives in clinical practice (6, 7). However, the use of oral chloral hydrate has become limited due to its gastrointestinal effects in children (8), and propofol requires close monitoring of vital signs, complicating its use (9). Therefore, sedation strategies for young children must prioritize safety, non-invasiveness, and ease of use to enhance acceptance by both children and their guardians. Midazolam and dexmedetomidine are two frequently employed sedatives with distinct pharmacological properties that make them valuable in clinical settings. Midazolam, a benzodiazepine, is known for its rapid onset and short half-life, making it suitable for short-term sedation during surgical and diagnostic procedures (10). Dexmedetomidine (DEX), as a selective *α*2-adrenergic agonist, is very popular in clinical applications due to its sedative and analgesic properties (11). Nonetheless, more investigation is required to completely comprehend the efficacy and safety of combining these two drugs, especially in pediatric patients.

Although there is substantial research on the use of midazolam and dexmedetomidine for sedation in adult, studies focusing on their use in pediatric patients are relatively limited. Furthermore, optimizing the effect and safety of their combined use in clinical practice remains controversial. This study aims to address these gaps by investigating the combined use of midazolam and dexmedetomidine for sedation during pediatric MRI examinations. We seek to provide a more robust sedation protocol for clinical practice. Our goal is to precisely evaluate the efficacy and security of this combination through a prospective, randomized controlled trial and to validate its applicability in pediatric MRI settings using actual clinical data.

## 2 Materials and methods

### 2.1 Study design

The design of this investigation was randomized controlled trial with a prospective design. The study protocol received approval from our hospital's Ethics Committee and adhered to the ethical principles for child-related medical research outlined in the Declaration of Helsinki. Every parent or legal guardian of a child included in the trial received complete information on the procedure and gave signed informed consent. This study was approved by the Shanxi Children's Hospital Medical Ethics Committee (Approval Number: IRB-KYYN-2021-003), and conducted in Shanxi Children hospital from January 2022 to December 2023.

### 2.2 Study subjects

The study included 196 pediatric patients who underwent MRI examinations. Two groups of 98 individuals each were randomly assigned to the patients. The following were the inclusion criteria:

(1) Age between 1 month and 6 years, with an MRI examination duration ≤60 min.
(2) No serious cardiopulmonary disease or other systemic diseases.
(3) No history of allergy to midazolam or dexmedetomidine.
(4) Physical Condition Score (12) of I or II from the American Society of Anesthesiologists (ASA).

**Figure 1:**
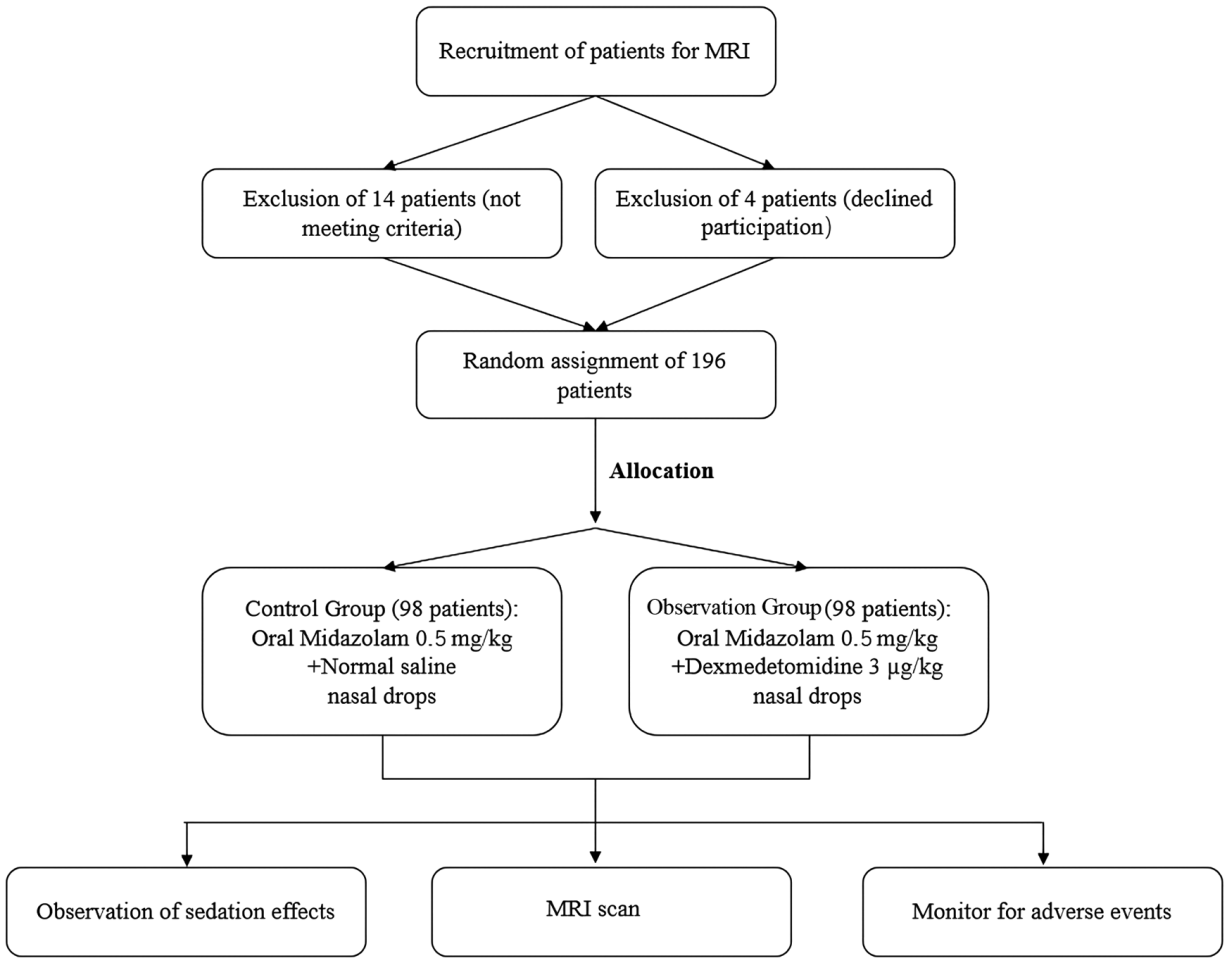
Study flow chart.

Exclusion criteria include:

(1) Severe psychological disorders or mental illness.
(2) History of sedatives and hypnotic drug use within the past 48 h.
(3) Body mass index (BMI) ≥28.
(4) History of allergy to the study drugs. In Figure 1, the experimental protocol is presented.

### 2.3 Randomization and blinding

Randomization was performed using a computer-generated randomization table to assign participants to two groups in a 1:1 ratio. Randomization codes were sealed in opaque envelopes and opened sequentially before intervention by the administering anesthesiologist. To ensure blinding, the sedation nurse, data collectors, and outcome assessors were unaware of group assignments. The grouping aimed to evaluate the added effect of intranasal dexmedetomidine on sedation success and safety in pediatric MRI sedation.

This study was designed as a single-blind randomized controlled trial. Participants, their guardians, and the outcome assessors were blinded to group assignments. To maintain blinding, the intranasal dexmedetomidine (active drug) and saline placebo were prepared in identical containers with matching volumes and administered using the same technique. The administering anesthesiologist was not blinded due to the nature of the intervention but was not involved in data collection or outcome assessment. All staff responsible for data collection, analysis, and reporting remained blinded to the group assignments throughout the study.

### 2.4 Grouping and intervention

Prior to sedation: All children adhered to standardized fasting guidelines (13). For a duration of 2 h, only clear fluids were allowed, followed by 4 h of breast milk, 6 h of bland meals or baby formula, and 8 h of meat or fatty or fried foods. On the day the MRI was performed, children were allowed to take their usual necessary medications with small amounts of clear fluids. There were no specific requirements regarding the duration of wakefulness prior to sedation. Before administering medication, baseline vital signs were recorded, including blood oxygen saturation (SpO_2_), heart rate (HR), and blood pressure (BP).

Sedation process: The control group (CG) [oral midazolam (OM) group] received an oral dose of midazolam solution (2 mg/ml, batch number 0L912011, Yichang Renfu Pharmaceutical, Yichang, China) at 0.5 mg/kg. The sedation nurse, along with the child's guardia assisted the child in taking the medicine. In this group, a normal saline nasal drop of equal volume was administered in place of the dexmedetomidine used in the OG. The OG (midazolam combined with dexmedetomidine nasal drops group) received OM solution at 0.5 mg/kg of dose and dexmedetomidine nasal drops (0.1 mg/ml, batch number 21033131, Yangtze River Pharmaceutical Group, Taizhou, China) at a dose of 3 μg/kg. After the child ingested the midazolam solution, the dexmedetomidine solution was administered in two equal amounts through both nostrils. Following drug administration, the child was positioned supine, and the nasal cavity was rubbed gently for 1–2 min to make sure complete absorption of the medication.

After medication: record the onset time of sedation, awakening time, possible adverse reactions, and total sedation time. The Ramsay scale was used to gauge the level of sedation (Table 1) (14), repeated every 10 min.

a. Successful sedation: Sedation was considered successful when the Ramsay Sedation Score was ≥5 points. At this level, the MRI examination was conducted, with the child entering the machine and completing the examination in a single session.
b. Failed sedation: Sedation was deemed unsuccessful if the onset time exceeded 30 min, the Ramsay Sedation Score was <5 points, or if the child woke up during the examination. In cases of failed sedation, the child was transferred to the recovery room for observation. Discharge from the recovery room was permitted when the Modified Aldrete Score (MAS) reached ≥9 points (Table 2) (15).

**Table 1:** Ramsay sedation score.

Score	Response
1	Awake, anxious, agitated, restless
2	Awake, cooperative, tranquil
3	Responds to commands
4	Asleep, brisk response to stimulus
5	Asleep, sluggish response to stimulus
6	Asleep, no response to stimulus

**Table 2:** Modified Aldrete score.

Score	Response
Breathing	
2	Able to breathe deeply
1	Dyspnea
0	Apnea
Circulation	
2	Systemic blood pressure <20% of the preanesthetic level
1	Systemic blood pressure between 20% and 49% of the preanesthetic level
0	Systemic blood pressure ≥50% of the preanesthetic level
SpO_2_	
2	Maintaining O_2_ saturation >92% on room air
1	Needing inhalation to maintain O_2_ saturation >92%
0	O_2_ saturation <92% despite O_2_ supplementation
Consciousness	
2	Fully awake
1	Arousable
0	Not responding
Mobility	
2	Able to move four extremities on command
1	Able to move two extremities on command
0	Able to move zero extremities on command

**Figure 2:**
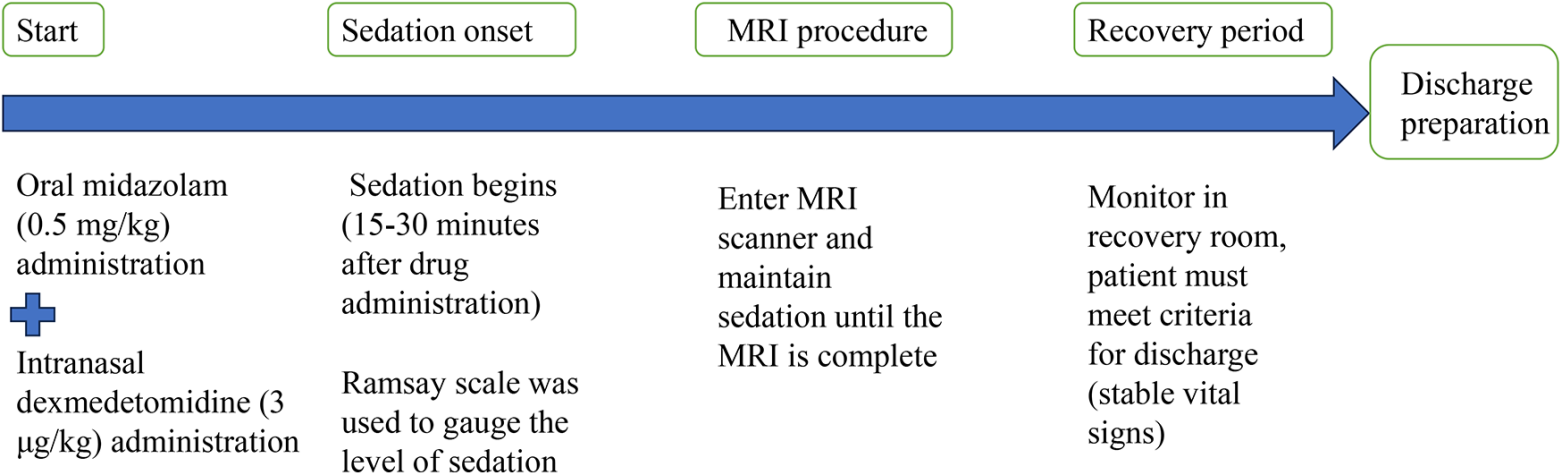
A flow diagram illustrates the timing of drug administration, the onset of sedation, MRI procedure, and recovery period.

A flow diagram illustrated the timing of drug administration, the onset of sedation, MRI procedure, and recovery period (Figure 2). This diagram aims to provide other centers with a clear and structured protocol for implementing this sedation approach.

### 2.5 Observation indicators

The following facts and information were documented: (1) The demographic characteristics of the two groups of children, which include gender, age, and body mass index. (2) Pre- and post-drug administration vital indicators, such as blood oxygen saturation (SpO2), heart rate (HR), and blood pressure (BP). (3) Sedation parameters: The sedation onset time refers to the duration from the administration of the medication to when the Ramsay Sedation Score reaches or exceeds 5. Awakening time is the duration from drug administration to when the MAS (Motor Activity Assessment Scale) reaches or exceeds 9. Total sedation time is the duration from drug administration to when the Modified Aldrete Score reaches or exceeds 9. Furthermore, the success rate of sedation on a single occasion was determined by calculating the percentage of patients who stayed motionless throughout the whole MRI procedure and finished it successfully. (4) Incidence of adverse reactions: including changes in HR higher than 20% from baseline, changes in BP higher than 20 mmHg from baseline, SpO2 < 90%, and occurrences of nausea and vomiting.

### 2.6 Sample size

Based on the results of a pilot study, the expected sedation success rates were 50% in the control group (oral midazolam alone) and 80% in the observation group (oral midazolam combined with intranasal dexmedetomidine). A sample size calculation was performed using the formula for comparing two proportions. Assuming a significance level of 0.05 and a power of 80%, the required sample size was calculated to be 34 patients per group. Considering a 10% drop-out rate, a sample size of 38 patients per group will be appropriate. This sample size (*n* = 96/group) would provide sufficient power to detect a significant difference in sedation success rates between the two groups.

### 2.7 Statistical analysis

All data were collected by trained researchers and recorded using standardized forms. The information was shown as a percentage or as mean ± standard deviation. The independent samples *t*-test was used to assess differences between groups for continuous variables, while the chi-square test was employed for categorical data. The statistical analysis was performed using SPSS version 25.0. Two-sided tests were done with a significance level of *p* < 0.05.

## 3 Results

### 3.1 General information between the two groups of patients

Between January 2022 and December 2023, a total of 214 children aged from one month to six years old who underwent MRI examinations were enlisted. After screening, 14 children were excluded due to their failure to meet the criteria for inclusion, and the other 4 children chose to withdraw from the study. Therefore, 196 children were allocated at random to the CG and the OG according to the principle of 1:1, with each group containing 98 children. There were no discernible changes involving the two groupings in general information such as age, gender ratio, weight, ASA classification, and MRI examination duration (*P* > 0.05, Table 3), suggesting that the experimental design was random. The allocation was reasonable and effective, and the two groups of children were well comparable at baseline (Table 3).

**Table 3:** Demographic data of the individuals (*n* = 196).

Group	Control-group (*n* = 98)	Observation-group (*n* = 98)	*t*/*χ*^2^	*P*
Male/female	48/50	56/42	1.281	0.258
Age (month)	29.01 ± 16.59	28.00 ± 17.35	0.351	0.725
ASA(Ⅰ/Ⅱ)	88/10	91/7	0.576	0.448
Weight (kg)	12.88 ± 3.41	13.02 ± 3.89	0.589	0.557
Duration of examination (min)	37.56 ± 10.55	38.35 ± 11.02	0.641	0.521
MRI examination sites classification				
Brain	69 (70.4%)	71 (72.4%)		
Chest	8 (8.2%)	7 (7.1%)		
Abdomen	7 (7.1%)	6 (6.1%)		
Lumbar	4 (4.1%)	5 (5.1%)		
Biliary	2 (2.0%)	2 (2.0%)		
Urinary tract system	3 (3.1%)	3 (3.1%)		
Limbs	3 (3.1%)	2 (2.0%)		
Spinal cord	2 (2.0%)	2 (2.0%)		
Diagnostic categories				
Neurosurgery	20 (20.4%)	22 (22.4%)		
Neurobehavioral disease	15 (15.3%)	12 (12.2%)		
Motor/language retardation	10 (10.2%)	9 (9.2%)		
Epilepsy	8 (8.2%)	9 (9.2%)		
Hepatitis syndrome	5 (5.1%)	6 (6.1%)		
Urology	7 (7.1%)	7 (7.1%)		
Endocrinology	5 (5.1%)	5 (5.1%)		
Orthopedics	10 (10.2%)	8 (8.2%)		
Others	18 (18.4%)	20 (20.4%)		

The data are shown as mean ± standard deviation (SD) and all had a normal distribution. Counts are used to illustrate categorical data.

### 3.2 Sedative effect

We analyzed the awakening time, sedation onset time, and total sedation time for both groups. There was no considerably distinction in the sedation onset time and awakening time involving the two groupings (*P* > 0.05). However, the total sedation time was significantly longer in the observation group (OG) (87.46 ± 25.84 min) compared to the CG (80.46 ± 15.95 min) (*P* = 0.039). The one-time sedation success rate was also assessed: 78.57% in the CG vs. 87.96% in the OG. The midazolam combined with dexmedetomidine nasal drops group demonstrated a higher one-time sedation success rate compared to the midazolam-only group, with this difference being considerably (*P* = 0.038, Table 4).

**Table 4:** Sedative effect of the two groups (*n* = 196).

Group (*n* = 98, each group)	Sedation onset time (min)	Recovery time (min)	Overall sedation time (min)	Success rate (%)
Control-Group	22.67 ± 15.86	58.34 ± 20.45	80.46 ± 15.95	78.57
Observation-Group	20.65 ± 16.82	55.56 ± 22.59	87.46 ± 25.84	87.96
*t*/*χ*^2^	0.896	0.783	−2.087	2.072
*P*	0.371	0.435	0.039	0.038

The data are shown as mean ± standard deviation (SD) and all had a normal distribution. Counts are used to illustrate categorical data.

To further compare the sedative effects of the two regimens, Ramsay sedation scores were noted at five time points: T0 (before medication), T1 (10 min after medication), T2 (upon falling asleep), T3 (at the end of the examination), and T4 (upon waking). All data were normally distributed. The comparison of Ramsay scores between the control and observation groups at these time points is detailed as follows:

**T0 (Before administration):** No discernible change in Ramsay scores was observed involving the two groupings before medication (*P* = 0.634).
**T1 (10 min after administration):** The OG exhibited a considerably higher sedative effect in contrast to the CG (*P* = 0.005).
**T2 (Upon Falling Asleep):** The sedation effect of the OG was considerably superior to that of the CG during the falling asleep phase (*p* = 0.010).
**T3 (At the end of the examination):** The Ramsay score for the OG at the end of the examination was still considerably higher than that for the CG (*P* = 0.029).
**T4 (Upon Waking):** No discernible change in Ramsay scores involving the two groupings upon wakening (*P* = 0.197).

**Figure 3:**
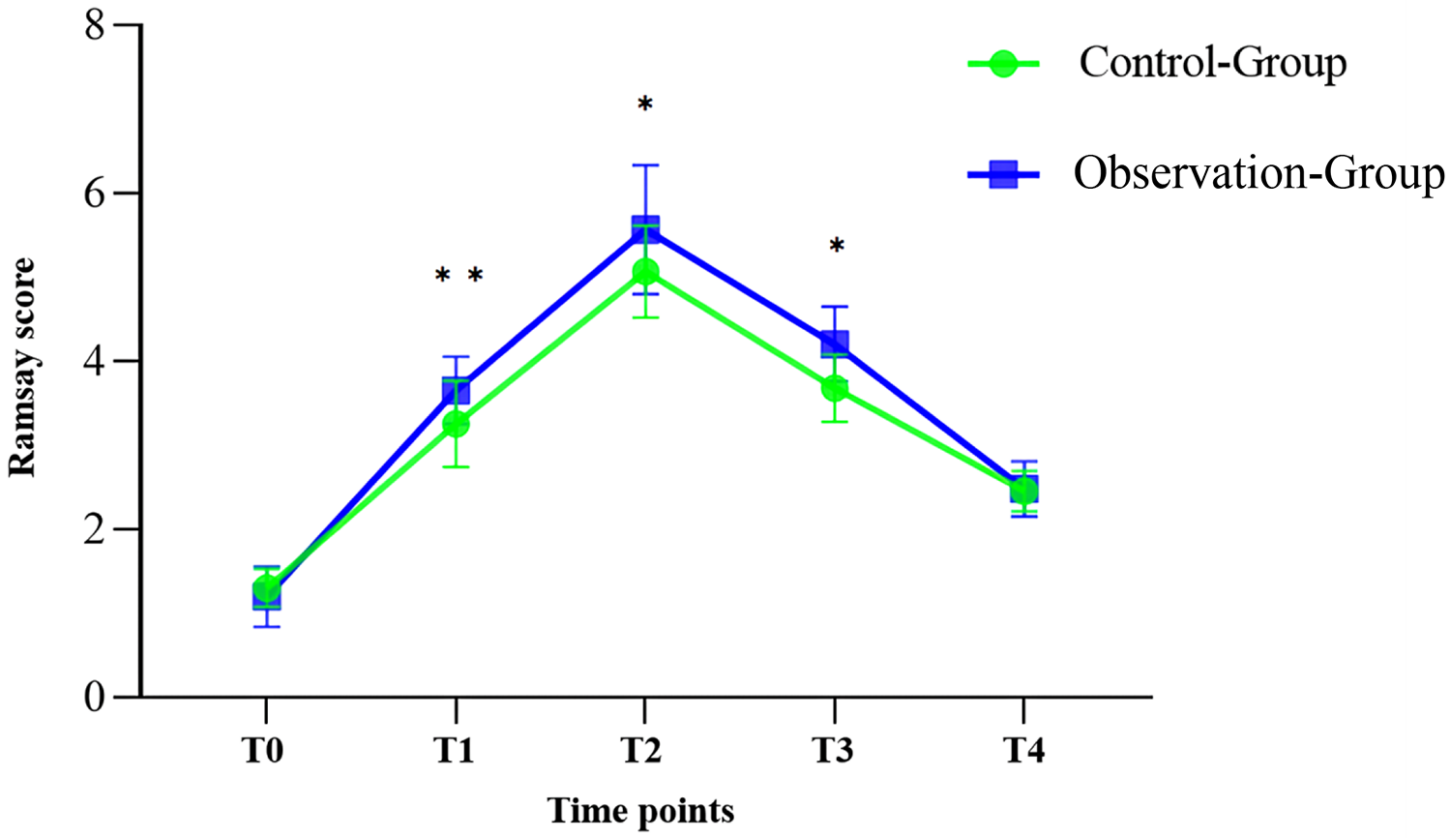
Ramsay scores at each time point in the two groups. All data are Normally distributed. The error bars in the figure represent the standard deviation of each group. Significant differences are marked with asterisks.

These results indicate that the sedation effect of the OG was considerably better after medication, during the process of falling asleep, and at the end of the examination. However, there weren’t any notable variations between the groups before administration and upon waking. This data is illustrated in Figure 3.

### 3.3 Adverse events

We compared the different rates of adverse events between the two groups. HR after sedation decreased by 20% compared with that before sedation in 6 cases in CG and 5 cases in OG. BP after sedation was reduced by 20% compared with that before sedation, 4 cases in the CG and 2 cases in the OG. In addition, adverse events including hypoxia, delirium, agitation, discoordination, and drowsiness were recorded. The above data showed that there were not any notable variations in the incidence of these adverse events involving the two groupings (*P* > 0.05), as shown in Table 5. None of the 27 patients with adverse events received specific intervention, and all adverse event symptoms were resolved after rest.

**Table 5:** Adverse events of the two groups.

Variables	Control-group	Observation-group	*t*/*χ*^2^	*P*
Abnormal HR	6 (6.1%)	5 (5.1%)	0.091	0.763
Abnormal BP	4 (4.1%)	2 (2.0%)	0.693	0.405
Anoxia	0	0	—	—
Aelirium	1 (1.0%)	0	1.010	0.315
Nausea and vomiting	4 (4.1%)	2 (2.0%)	0.693	0.405
Agitation	1 (1.0%)	1 (1.0%)	0.000	1.000
Loss of coordination	0	0	—	—
Lethargy	0	1 (1.0%)	1.010	0.315

## 4 Discussion

This research assessed the safety and sedative efficacy of midazolam with intranasal dexmedetomidine (ID) during pediatric MRI exams using a prospective, randomized controlled trial design. From the perspective of sedation efficacy, the combination of midazolam with dexmedetomidine intranasal drops demonstrated no significant difference from midazolam alone in terms of sedation onset time and recovery time. However, the total sedation time for the OG was considerably longer than that for the CG (87.46 ± 25.84 min vs. 80.46 ± 15.95 min). This indicates that the combined medication provides a more prolonged sedative effect while maintaining effective sedation.

Additionally, the one-time sedation success rate was considerably higher in the OG in contrast to the control group (87.96% vs. 78.57%), further highlighting the superiority of combining midazolam with dexmedetomidine for pediatric MRI examinations. The Ramsay sedation score data also reveal that the sedation effect in the OG was considerably better than in the control group at 10 min post-administration (T1), upon falling asleep (T2), and at the end of the examination (T3). There were no discernible variations involving the groupings before administration (T0) and upon awakening (T4). Clinically, this suggests that the addition of intranasal dexmedetomidine to oral midazolam enhances the depth of sedation, as reflected by the higher scores in the observation group. A higher Ramsay score indicates a deeper and more stable sedation level, which is particularly beneficial in procedures requiring prolonged immobility, such as MRI scans. The increased sedation effectiveness in the observation group potentially reduces the need for additional sedation or adjustments, leading to a more efficient and comfortable experience for both patients and healthcare providers. These findings suggest that the addition of dexmedetomidine significantly enhances the sedative effects of midazolam, particularly during the initial stages of sedation and throughout the examination, thereby providing a more stable and effective sedation state. These results highlight the clinical advantage of combining midazolam with dexmedetomidine in pediatric sedation protocols.

In terms of safety, this study meticulously recorded changes in vital signs and adverse events in both groups of children during the sedation process. The results indicated no discernible change in the incidence of adverse events involving the two groupings, and no significant negative occurrences were noted. All adverse symptoms were resolved with rest, suggesting that the combination of OM with ID is a safe sedation regimen. Specifically, heart rate reductions of 20% from baseline were observed in 6 cases (6.1%) in the control group and 5 cases (5.1%) in the OG, with no notable variation involving the groupings. Similarly, there wasn’t a noticeable variation in the incidence of blood pressure reductions by 20% or in adverse events such as nausea and vomiting between the two groups. These data suggest that while the combined sedation regimen may prolong the total sedation time, it doesn't significantly increase the risk of adverse events.

Midazolam, a commonly used short-acting benzodiazepine sedative, is favored for its rapid onset and quick metabolism, making it a popular choice in clinical practice (16). However, monotherapy with midazolam may present challenges such as inadequate sedation depth and short duration. Dexmedetomidine, a selective *α*2-adrenergic agonist, offers a stable sedative effect and does not compromise respiratory function, making it increasingly recognized in pediatric sedation (17, 18). The findings of this investigation show that combining intranasal midazolam with dexmedetomidine not only extends the sedation duration but also increases the success rate of achieving effective sedation on the first attempt, without a significant rise in adverse events. This combination regimen presents several advantages for clinical practice (19). Firstly, nasal administration is more convenient and less invasive compared to intravenous routes, which enhances acceptance among children and their guardians. Secondly, the inclusion of dexmedetomidine not only improves the overall sedative effect but also allows for a reduced dosage of midazolam, thereby minimizing potential side effects. Lastly, the simplicity of this approach, which does not require complex equipment or procedures, facilitates its adoption in medical settings with limited resources (20). Based on the findings of this study, we recommend that centers implementing this combined sedation approach adopt rigorous monitoring protocols to ensure patient safety. Continuous monitoring of vital signs, including SpO_2_, HR, and BP, is essential throughout the sedation process. Additionally, the Ramsay Sedation Score should be regularly assessed to ensure that sedation depth is maintained appropriately for the procedure. Although all adverse events were resolved with rest in this study, we recommend that healthcare providers be prepared to intervene if necessary. In the event of significant respiratory depression or cardiovascular instability, immediate interventions, including oxygen supplementation or pharmacological reversal agents, should be available. The sedation regimen should be interrupted promptly if sedation depth exceeds safe levels, and the patient should be monitored until full recovery is confirmed.

When comparing our findings to other pediatric sedation protocols currently in use, several factors related to cost-effectiveness and resource utilization must be considered. While the combination of oral midazolam and intranasal dexmedetomidine may incur higher upfront drug costs compared to traditional agents such as chloral hydrate or propofol, it may offer significant savings in terms of resource utilization. The reduced incidence of sedation failures and adverse events in our study suggests that this combined sedation regimen may lead to fewer repeat procedures or additional interventions, ultimately saving time and reducing the overall burden on healthcare resources. Moreover, dexmedetomidine's minimal respiratory depression could reduce the need for intensive monitoring or emergency interventions, potentially lowering hospital costs. In comparison to other protocols that require intravenous access or more invasive methods, the non-invasive nature of intranasal dexmedetomidine could reduce the need for intravenous catheters, thus decreasing procedural costs and patient discomfort. Furthermore, by achieving a higher sedation success rate with fewer adverse events, this protocol may contribute to more efficient use of anesthesia staff and recovery room resources.

While this study provides substantial evidence supporting the use of OM combined with ID for pediatric MRI examinations, there are notable limitations. First off, selection bias may be introduced and the applicability of the findings may be limited by the study's single-center design and very small sample size. Future research should involve multicenter trials with larger sample sizes to enhance the applicability and reliability of the results. Secondly, this study focused on evaluating the sedation effect and immediate adverse events but did not assess long-term safety. Future investigations should incorporate long-term follow-up to comprehensively assess the sustained effects and potential risks associated with this sedation regimen. Finally, intranasal administration, while minimally invasive and generally well-accepted, has limitations such as variable absorption rates due to individual differences in nasal anatomy or physiological conditions (e.g., nasal congestion or inflammation). Additionally, patient cooperation during administration may pose a challenge, particularly in pediatric populations. To mitigate these issues, we adopted a standardized administration protocol, which included dividing the dose equally between nostrils and ensuring proper positioning for 1–2 min to enhance absorption. Parental presence and calming techniques were also employed to improve patient cooperation. Despite these measures, we acknowledge that these factors could introduce variability in the sedation outcomes.

## 5 Conclusion

This study preliminarily confirmed the sedation effect and safety of midazolam combined with dexmedetomidine intranasal drops in pediatric MRI examinations, providing an efficient, convenient, and safe sedation regimen. With further in-depth research and accumulation of evidence, it is believed that this regimen will be more widely used in clinical practice.

## Data Availability

The original contributions presented in the study are included in the article/Supplementary Material, further inquiries can be directed to the corresponding author.
